# The Use of a Penta-Deuterophenyl Substituent to Improve the Metabolic Stability of a Tyrosine Kinase Inhibitor

**DOI:** 10.3390/molecules29246042

**Published:** 2024-12-22

**Authors:** Júlia Dulsat, Raimon Puig de la Bellacasa, José I. Borrell

**Affiliations:** Grup de Química Farmacèutica, IQS School of Engineering, Universitat Ramon Llull, Via Augusta 390, E-08017 Barcelona, Spain; juliadulsatm@iqs-blanquerna.url.edu (J.D.); raimon.puig@iqs.url.edu (R.P.d.l.B.)

**Keywords:** metabolism, deuteration, pyrido[2,3-*d*]pyrimidin-7(8*H*)-one, tyrosine kinase inhibitor

## Abstract

In cases in which a rapid metabolism is the cause of an unfavorable pharmacokinetic profile, it is important to determine the Sites of Metabolism (SoMs) of a molecule to introduce the necessary modifications to improve the stability of the compound. The substitution of hydrogen atoms by deuterium atoms has been proposed to ameliorate such properties due to the greater stability of the C-D bonds. **IQS016**, bearing a 2-phenylamino substituent, is a compound previously described by our group with good biological activity as a discoidin domain receptor (DDR2) inhibitor but suffers from low metabolic stability determined in a test with rat-liver microsomes (less than 50% of the initial compound after 60 min). We have obtained the corresponding 2-(penta-deuterophenyl) analog (**IQS016-*d_5_***) from aniline-2,3,4,5,6-*d_5_* showing that it has a better metabolic stability than **IQS016** and a higher inhibitory effect on isolated tyrosine kinase receptors but not a better 2D in vitro effect.

## 1. Introduction

The physicochemical properties and the pharmacokinetic profile play a key role in giving a chemical compound the status of a drug candidate. For this reason, it is important to study them in the initial stages of drug development. Concerning metabolism, studying the behavior of a compound allows us to know, on the one hand, its stability over time and, on the other, the identity of the main metabolites to characterize them both at a pharmacological and toxic level. This knowledge can be used as a starting point in the process of the optimization and improvement (“hit to lead”) of the chemical structure of the desired compound [1].

In cases of unfavorable pharmacokinetics due to too-fast metabolism, it is important to know the Sites of Metabolism (SoMs) of the molecule to modify the lead compound to improve metabolic stability, thus reducing the drug dose and administration frequency [2]. Considering that the most common pathway for the elimination of a drug is the oxidative metabolism by P450 cytochromes (CYPs) [3], one typical approach used to reduce CYP metabolism is to block the most prone positions for the oxidation of the molecule [4]. The most common approach for this aim is the introduction of functional groups that make difficult the oxidation at such positions, such as alkoxy groups, substituted amines, and, more frequently, fluorine atoms [5,6,7]. However, the wide use of fluorine in Medicinal Chemistry (45% of the drugs approved by the FDA in the period 2018–2019 contained fluorine) has started to be controversial [8] due to the potential toxicity of the fluorine anion released in substitution reactions of the C-F in which the fluorine atom can act as a leaving group. Furthermore, such substitution reactions can produce toxic metabolites of the parent compound.

An alternative strategy consists of the replacement of the hydrogens present in those positions easily oxidizable by CYPs by deuterium [9,10,11]. Such a substitution usually has minimal effects on the structure, physical properties, and pharmacology of the resulting compound in comparison with the non-deuterated parent one due to the similar characteristics of hydrogen and deuterium. However, in some cases, a significant effect on the pharmacokinetics has been observed due to the so-called “deuterium isotope effect” that can lower the rate of C-D versus C-H bond cleavage, thus rendering unpredictable the effect of CYP450 on the oxidation of such positions [11]. Even so, in 2017, the FDA approved Austedo (deutetrabenazine), the first deuterated drug for the treatment of Huntington’s disease [12].

**Figure 1:**
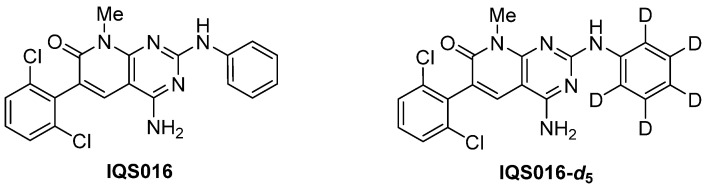
Structures of **IQS016** and the corresponding deuterated analog **IQS016-*d_5_***.

As a part of our research in the field of tyrosine kinase inhibitors (TKIs), we described the compound **IQS016** [13] (Figure 1) that was later licensed as a part of a project focused on the squamous cell carcinoma of the lung (SCC) presenting good antitumor activity as a discoidin domain receptor (DDR2) inhibitor [14,15]. The major drawback of **IQS016** was its metabolic instability as it was established by an in vitro stability study and metabolism profiling carried out at Hepatochem (https://hepatochem.com/, accessed on 3 September 2024) using rat-liver microsomal incubation followed by LS-MS analysis. Such a study showed that after 60 min of incubation, less than 50% of **IQS016** remained intact and the most important SoMs were present at the 2-phenylamino substituent (unpublished results).

More recently, as a part of a project on pancreatic cancer, we decided to use the deuteration approach and synthesize the **IQS016-*d_5_*** analog (Figure 1) to evaluate whether such a deuterated compound improves metabolic stability, solving the pharmacokinetic problems of **IQS016** but maintaining the biological activity of **IQS016**.

The introduction of the penta-deuterophenyl group in **IQS016-*d_5_*** would be achieved by using aniline-2,3,4,5,6-*d_5_* as a precursor as it was already used in the case of atorvastatin [16]. The present paper deals with the synthesis of **IQS016-*d_5_*** and the results obtained in the study of its metabolic stability and biological properties.

## 2. Results and Discussion

We have recently shown that there are a large number of free online available tools for the prediction of the ADME properties (Absorption Distribution, Metabolism, and Excretion) of a drug candidate [17]. In the case of metabolism, the capabilities of such resources range from the prediction of the most probable SoMs (such as in the case of the very useful SMARTCyp tool [18,19,20]) to the structure of the resulting metabolites (such as in BioTransformer 3.0 [21,22]). However, in the case of deuterated compounds, to the best of our knowledge, only the software Molecular Forecaster (Version 6383) [23,24] (academic licenses are free of charge) allows the prediction of SoMs and the structures of possible metabolites of compounds containing C-D bonds. IMPACTS (In silico Metabolism Prediction by Activated Cytochromes and Transition States) is the module responsible for such calculations by modeling the CYP-drug transition states that return as SoMs, the reactive points closest to the oxygen bound to the iron atom of the heme group. Unlike other platforms, Molecular Forecaster includes, in its calculations, the reactivity, the ligand–protein binding affinity, and the geometry of the transition state to generate a result.

**Figure 2:**
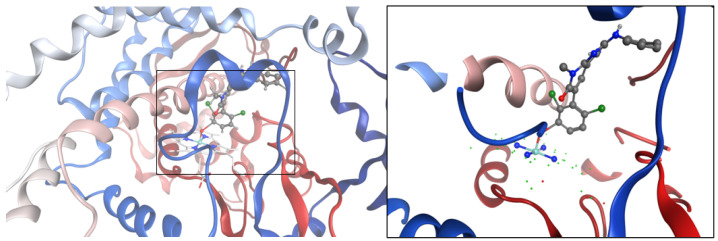
Graphical representation of the drug–enzyme interaction between **IQS016-*d_5_*** and the heme group of the 2C8CYP enzyme obtained from the Molecular Forecaster software.

When we used such tools with **IQS016**, they clearly pointed out the 2-phenylamino substituent as the most favorable place for oxidation by the P450 cytochromes. On the contrary, the use of Molecular Forecaster in **IQS016-*d_5_*** proposes, as a SoM, the *m*-position of the 6-(2,6-dichlorophenyl) substituent, the 4-amino group, and the N8-methyl group in agreement with the expected lower reactivity of the C-D bonds. Thus, Figure 2 shows the representation of the drug–enzyme interaction between the most likely SoM of **IQS016-*d_5_*** (the *m*-position of the 6-dichlorophenyl substituent) with the Fe^2+^ atom of the heme group of the 2C8CYP enzyme as predicted by the IMPACTS module of the Molecular Forecaster software.

**Scheme 1:**
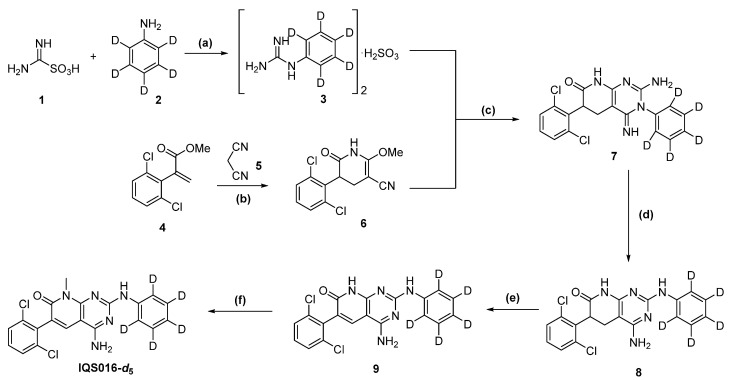
Synthetic route for **IQS016-*d_5_***: (a) MeOH, MW irradiation, 65 °C, 2 min, 79%; (b) NaOMe, MeOH, 5 h, reflux, Ar atmosphere, 99%; (c) (1) **3**, anhydrous 1,4-dioxane, NaOMe, 65 °C, 15 min and (2) addition of **6**, 140 °C, 40 min, 79%; (d) NaOMe, MeOH, 140 °C, 40 min, 78%; (e) anhydrous DMSO, NaH, 10 min room temp., 100 °C for 4 h, 79%; (f) NaH, anhydrous DMSO, MeI, room temp., overnight, 68%.

With this information in hand, we synthesized **IQS016-*d_5_*** using the synthetic route included in Scheme 1, which is the same used for the synthesis of **IQS016** but using aniline-2,3,4,5,6-*d_5_* as a precursor [13].

**Figure 3:**
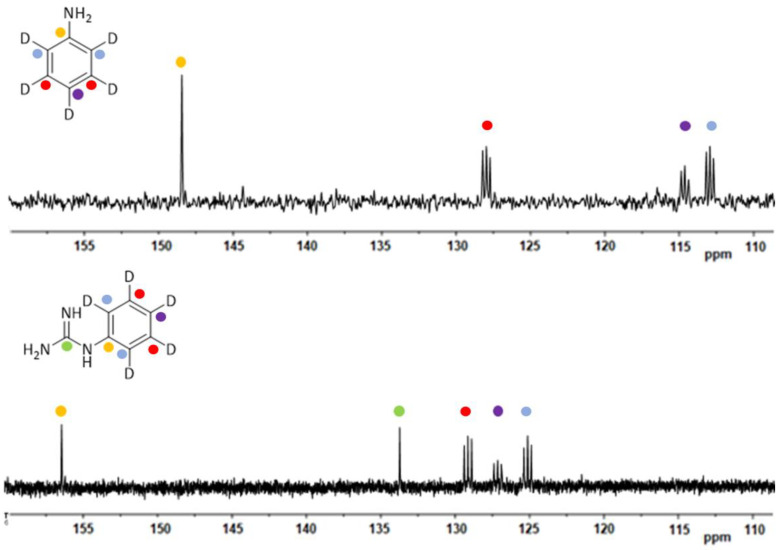
^13^C-NMR spectra (D_2_O) of aniline-2,3,4,5,6-*d_5_* (**2**) and 1-(phenyl-*d_5_*)guanidine hydrogen sulfite (**3**).

First, we prepared the non-previously described 1-(phenyl-*d_5_*)guanidine hydrogen sulfite (**3**) upon the treatment of AIMSOA (**1**, aminoiminomethanesulfonic acid, obtained in 86% yield by the reaction of formamidinesulfinic acid with 39% peracetic acid solution in acetic acid at 20 °C for 5 h [25]) with aniline-2,3,4,5,6-*d_5_* (**2**) in MeOH at 65 °C under microwave irradiation for 5 min in 79% yield. To monitor the reaction, the ^13^C-NMR spectra of both the aniline-2,3,4,5,6-*d_5_* (**2**) and the resulting guanidine were recorded (Figure 3). In the case of **2**, the spectrum showed four signals: a singlet at 148.3 ppm, corresponding to the C-NH_2_, and three triplets at 128.3, 115.0, and 113.3 ppm with relative intensities of 2:1:2, which correspond to the two *meta*, the *para*, and the two *ortho* carbon atoms coupled with the deuterium atoms. This pattern was also present in the ^13^C-NMR spectrum of the 1-(phenyl-*d_5_*)guanidine hydrogen sulfite (**3**) although the carbon signals of the phenyl-*d_5_* ring were displaced due to the formation of the guanidine in the form of hydrogen sulfite salt, and a new signal appeared at 133.9 ppm, corresponding to the guanidine carbon (Figure 3).

Next, we synthesized 5-(2,6-dichlorophenyl)-2-methoxy-6-oxo-1,4,5,6-tetrahydropyridine-3-carbonitrile (**6**) by the reaction of 2-(2,6-dichlorophenyl)acrylate (**4**) [26] and malononitrile (**5**) in the presence of NaOMe/MeOH in an almost-quantitative yield [27]. The subsequent treatment of **3** with NaOMe in anhydrous 1,4-dioxane at 65 °C for 15 min to liberate the 1-(phenyl-*d_5_*)guanidine followed by the addition of **6** and the heating of the mixture at 140 °C for 40 min afforded the 2-amino-6-(2,6-dichlorophenyl)-4-imino-3-(phenyl-*d_5_*)-4,5,6,8-tetrahydropyrido[2,3-*d*]pyrimidin-7(*3H*)-one (**7**) in 79% yield.

To convert such a 3-(phenyl-*d_5_*) substituted isomer into the desired 2-(phenyl-*d_5_*)pyridopyrimidine, we used the Dimroth transposition already described by our group for the synthesis of the corresponding 2-phenyl derivative [27]. Thus, the treatment of **7** with NaOMe/MeOH at 140 °C for 40 min afforded **8** in 78% yield. Compound **8** was dehydrogenated in the presence of NaH in anhydrous DMSO at 100 °C for 4 h to afford 4-amino-6-(2,6-dichlorophenyl)-2-((phenyl-*d_5_*)amino)pyrido[2,3-*d*]pyrimidin-7(*8H*)-one (**9**) in 79% yield. Finally, **9** was methylated using MeI in the presence of NaH in DMSO to yield the desired 4-amino-6-(2,6-dichlorophenyl)-8-methyl-2-((phenyl-*d_5_*)amino)pyrido[2,3-*d*]pyrimidin-7(*8H*)-one (**IQS016-*d_5_***) in 68% yield.

Compounds **7**, **8**, **9**, and **IQS016-*d_5_*** were characterized by using ^1^H- and ^13^C-NMR spectroscopy together with HRMS. The detection in the ^13^C-NMR spectra of the phenyl-*d_5_* carbon atoms coupled with the deuterium atoms was virtually impossible because such signals were too weak (presenting also coupling with the deuterium atoms), but this fact permitted a better assignment of the rest of the carbons of the molecule.

**Figure 4:**
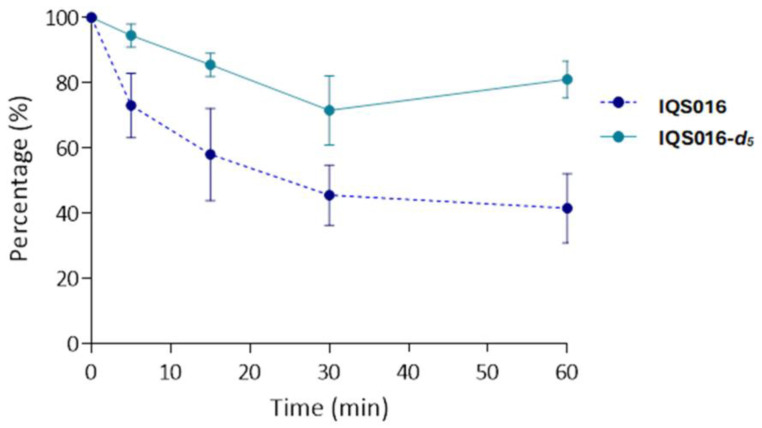
Metabolic stability of **IQS016** (n = 2) and **IQS016-*d_5_*** (n = 2) using the rat-liver microsome incubation protocol.

Once we obtained **IQS016-*d_5_***, we decided to study the compared in vitro metabolic stability of **IQS016** and **IQS016-*d_5_*** by using the rat-liver microsome incubation protocol [28,29], followed by the HPLC-MS/MS analysis of the incubation at 0, 5, 15, 30, and 60 min. As can be seen in Figure 4 and Table S1 (Supporting Information), while **IQS016** showed the very rapid metabolism already commented on, **IQS016-*d_5_*** remained almost stable during the 60 min incubation with an apparent reduction of around 10–15%.

Such a result clearly indicates that the substitution of the 2-phenylamino substituent by the 2-(phenyl-*d_5_*)amino one improved the metabolic stability of the molecule as desired, proving the effect of the deuterium atoms on the metabolism. Deuterium has a lower molar volume (0.140 cm^3^/mol), is less lipophilic (∆log P = −0.006), and offers some variation in the acidity constant (pKa), thus reducing the acid character of carboxylic acids and phenols while increasing the basicity of amines. In addition, the C-D bond distance is shorter (0.005 Å) and, with the increase in mass, makes it more stable [30].

Subsequently, we decided to evaluate the impact of such substitution on the kinase inhibition profile by sending **IQS016** and **IQS016-*d_5_*** to Reaction Biology (https://www.reactionbiology.com/, accessed on 16 September 2024) and determining the residual activity at a concentration of 10 µM of the compounds in front of a group of kinases relevant for our research projects (Table 1).

The results obtained show a high degree of interaction and affinity between **IQS016** and the kinases BTK, EGFR, FGFR1, and FGFR2 since the resulting residual activity was around or less than 20%. Therefore, it can be concluded that **IQS016** is a multitarget compound against some membrane TKs. As for **IQS016-*d_5_***, the residual activities obtained were, for all the evaluated TKs, lower than in the case of **IQS016,** indicating either that deuterium atoms generate much stronger and more stable interactions with proteins than hydrogen atoms or that the possible increase in solubility results in an increase in the number of interactions.

**Figure 5:**
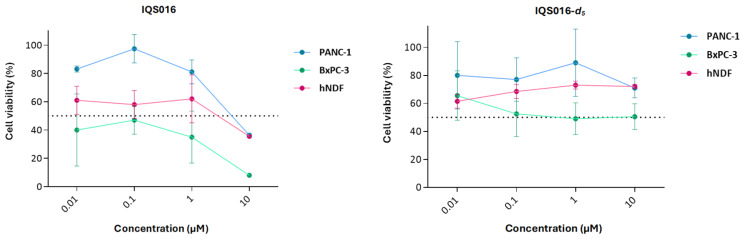
Cell viability (%) in a 2D culture of PANC-1 (blue), BxPC-3 (green), and hNDF (purple) after 72 h of incubation with **IQS016** and **IQS016-*d_5_*** (n = 3).

Such promising results brought us to evaluate, in classical 2D cell cultures, as part of a project centered on the use of our pyrido[2,3-*d*]pyrimidines as multitarget inhibitors, the biological activity of **IQS016** and **IQS016-*d_5_*** against non-carcinogenic cells (hNDF) and two human pancreatic ductal adenocarcinoma (PDAC) cell lines: BxPC-3 (epithelial phenotype) and PANC-1 (intermediate phenotype). Cell viability (Figure 5 and Table S2 in Supporting Information) was evaluated after 72 h of incubation with the compounds using an MTT (3-(4,5-dimethyl-2-thiazolyl)-2,5-diphenyl-2*H*-tetrazolium bromide) assay, a high-throughput screening test based on UV detection [31].

In the case of **IQS016** (Figure 5), the results show that at a concentration of 10 µM, the cell viability was lower than 50% for the three cell lines. The difference in behavior observed between PANC-1 and BxPC-3, both cancer cell lines, is particularly interesting: PANC-1 cells showed some resistance at the lower concentrations while for BxPC-3, the cell viability was less than 50% throughout the range. This difference could be explained by the invasive capacity of the PANC-1 cell line, which has a metastatic origin while BxPC-3 does not. Therefore, **IQS016** would offer greater sensitivity for those non-metastatic pancreatic cancers [32].

These results, combined with the improvement of the inhibitory profile of **IQS016-*d_5_*** with respect to **IQS016** shown above, brought us to expect a better result in the 2D biological activity of the deuterated analog. However, the results shown in Figure 5 pointed exactly to the contrary. Thus, the replacement of hydrogen by deuterium atoms leads to a decrease in the biological activity of **IQS016-*d_5_*** as a percentage of cell mortality higher than 50% was not reached across the whole range of concentrations tested. Only in the case of BxPC-3, the cell viability approached this value. Similar viabilities were obtained for hNDF and PANC-1 at high concentrations, PANC-1 being again the less sensitive cell line. These results, therefore, confirm the importance of the hydrogens present in the phenyl ring of **IQS016**.

Such discouraging results, together with the fact that as a part of our ongoing project on TKs against pancreatic cancer, we had identified a compound with similar metabolic stability to **IQS016-*d_5_*** but much better 2D and 3D biological activity [33], brought us to abandon **IQS016-*d_5_*** as a possible candidate for the treatment of such a disease. Nevertheless, the substitution of the 2-phenylamino group of **IQS016** by its deuterium analog achieved the initial objective of improving the metabolic stability of **IQS016-*d_5_***.

## 3. Materials and Methods

### 3.1. Synthesis of **IQS016-d_5_**

All solvents and chemicals were reagent-grade. Unless otherwise mentioned, all solvents and chemicals were purchased from commercial vendors and used without purification: Fluka (Honeywell Specialty Chemicals, Seelze, Germany), Sigma-Aldrich (Merck Life Science, Madrid, Spain), ABCR (Karlsruhe, Germany), and ACROS Organics (Thermo Fisher Scientific, Madrid, Spain). IR spectra were recorded in a Thermo Scientific Nicolet iS10 FTIR spectrophotometer (Thermo Fisher Scientific, Waltham, MA, USA) with Smart iTr. Wavenumbers (ν) are expressed in cm^−1^. ^1^H and ^13^C-NMR spectra were recorded on a Varian 400-MR spectrometer (Varian, Palo Alto, CA, USA) (^1^H-NMR at 400 MHz and ^13^C-NMR at 100.5 MHz). Chemical shifts were reported in parts per million (δ) and are referenced to dimethyl sulfoxide (DMSO-*d_6_*): 2.50 ppm in ^1^H-NMR and 39.5 ppm in ^13^C-NMR spectra. Coupling constants are reported in Hertz (Hz). Standard and peak multiplicities are designed as follows: s, singlet; d, doublet; dd, doublet of doublets; dt, doublet of triplets; t, triplet; q, quadruplet; quint, quintuplet; and br, broad signal. HRMS data were obtained by using a high-resolution mass spectrometer X500B QTOF system operating in ESI-positive mode (IQS Sciex Demo Lab). Elemental microanalyses were obtained on a EuroVector Instruments Euro EA elemental analyzer (EuroVector, Pavia, Italy). Microwave irradiation experiments were carried out in an Initiator^TM^ (Biotage, Uppsala, Sweden) microwave apparatus, operating at a frequency of 2.45 GHz with continuous irradiation power from 0 to 400 W. Reactions were carried out in 0.5, 2.5, 5, and 20 mL glass tubes, sealed with aluminum/Teflon crimp tops, which can be exposed up to 250 °C and 20 bar internal pressure. Temperature was measured with an IR sensor on the outer surface of the process vial. After the irradiation period, the reaction vessel was cooled rapidly to 50 °C by air jet cooling. The melting point was determined with a SMP3 melting point apparatus (Stuart Scientific). NMR spectra of final products are available in the Supplementary Materials.

Synthesis of 1-(phenyl-*d_5_*)guanidine hydrogen sulfite (**3**):

A quantity of 9.71 g (89.8 mmol) of formamidinesulfinic acid (Sigma-Aldrich, ref. F16001) was suspended in 30 mL of acetic acid. Then, 20 mL (120.0 mmol) of 39% peracetic acid solution was added dropwise to the suspension at room temperature. During the addition, temperature had to be maintained below 20 °C, so the process was performed in a water bath. After the addition was complete, the mixture was stirred at room temperature for 5 h. The resulting solid was filtered, washed with cold ethanol, and dried to finally afford 9.62 g (77.5 mmol, 86%) of AIMSOA (**1**, aminoiminomethanesulfonic acid) as a pure white solid. The spectroscopic data were consistent with those previously described [25]. mp: 122 °C. IR v_max_ (cm^−1^): 3235, 3019, 1682, 1433, 992, 730, and 648. Anal. Calc. for CH_4_N_2_SO_3_: C, 9.68%; H, 3.25%; N, 22.57%. Found: C, 9.73%; H, 3.17%; N, 22.50%.

In a 5 mL microwave vial, a mixture of 126 mg (1.0 mmol) of AIMSOA (**1**), 200 mg (2.0 mmol) of aniline-2,3,4,5,6-*d_5_* (Sigma-Aldrich, ref. 175692), and 5 mL of methanol was heated at 65 °C under microwave irradiation for 8 min. A clear solution was obtained and transferred to a round-bottom flask to remove the solvent under reduced pressure. Then, the resulting oil was heated at reflux with 25 mL of a 4:1 THF:CHCl_3_ mixture for 30 min. The organic layer was discarded and the treatment was repeated with fresh 25 mL of a 4:1 THF:CHCl_3_ mixture. During this process, a white solid appeared in the solution. Finally, the product was isolated by filtration and 580 mg (2.0 mmol, 79%) of the pure salt 1-(phenyl-*d_5_*)guanidine hydrogen sulfite (**3**) was finally obtained. mp: 243 °C. IR v_max_ (cm^−1^): 3545, 1668, 1614, 1554, 1505, 1457, 1388, 1092, and 1049. ^13^C-NMR (100.5 MHz, D_2_O) δ: 156.3 (s, *ipso*-C_6_D_5_), 133.9 (s, C=NH), 129.4 (1:1:1 triplet, *m*-C_6_D_5_), 127.4 (1:1:1 triplet, *p*-C_6_D_5_), and 125.5 (1:1:1 triplet, *o*-C_6_D_5_). HRMS (70 eV, ESI) (m/z) calculated for C_7_H_5_D_5_N_3_: 141.1188, [M + H]^+^; found: 141.1174, [M + H]^+^. Anal. Calc. for 2·C_7_H_4_D_5_N_3_·H_2_SO_3_: C, 46.37%; H, 2.79%; N, 23.18%. Found: C, 46.27; H, 3.01; N, 23.57.

Synthesis of 5-(2,6-diclorophenyl)-2-methoxy-6-oxo-1,4,5,6-tetrahydropyridine-3-carbonitrile (**6**):

A quantity of 1.13 g (20.91 mmol) of NaOMe was dissolved in 27 mL of anhydrous MeOH. Then, 1.90 g (15.7 mmol) of malononitrile (**5**) and 3.02 g (13.1 mmol) of methyl 2-(2,6-dichlorophenyl)acrylate (**4**) [26] were successively added and the mixture was heated at reflux for 5 h under argon atmosphere. Then, the solvent was removed under reduced pressure until a sponge-like solid was obtained. Finally, a minimum volume of water was added dropwise and the resulting solution was neutralized to pH 7 with a 2 M HCl solution. The solid formed was filtered and left to dry under vacuum for three days, affording 3.84 g (12.9 mmol, 99%) of **6** as a fine beige solid. Spectroscopic data were consistent with those previously described [27]. ^1^H-NMR (400 MHz, DMSO-*d_6_*) δ: 10.85 (s, 1H, N-H), 7.54–7.46 (m, 2H, *m*-H- C_6_H_3_Cl_2_), 7.36 (t, *J* = 8.1 Hz, 1H, *p*-H- C_6_H_3_Cl_2_), 4.68 (dd, *J* = 13.9, 8.4 Hz, 1H, C_5_-H), 3.95 (s, 3H, MeO), 2.92 (dd, *J* = 15.3, 14.0 Hz, 1H, C_4_-H), and 2.39 (m, 1H, C_4_-H).

Synthesis of 2-amino-6-(2,6-dichlorophenyl)-4-imino-3-(phenyl-*d_5_*)-4,5,6,8-tetrahydropyrido[2,3-*d*]pyrimidin-7(*3H*)-one (**7**):

A quantity of 1.105 g (3.0 mmol) of **3** was suspended in 15 mL of anhydrous 1,4-dioxane in a 20 mL microwave vial, then 230.65 mg (4.3 mmol) of NaOMe was added and the reaction mixture was heated at 65 °C for 15 min in an oil bath. After cooling down the mixture to room temperature, the white solid formed was filtered and washed with 5 mL of anhydrous dioxane. The filtrated liquid was transferred into a second 20 mL microwave vial containing 302.0 mg (1.0 mmol) of **6**. The vial was sealed and heated in an oil bath at 140 °C for 40 min. The resulting solution was transferred to a 100 mL round-bottomed flask and the solvent was removed under reduced pressure. A total amount of 327.3 mg (0.8 mmol, 79%) of pure **7** was obtained as a brown powder. mp: >300 °C. ^1^H-NMR (400 MHz, DMSO-d*_6_*) δ: 7.51 (ddd, *J* = 14.0, 8.1, 1.3 Hz, 2H, *m*-H- C_6_H_3_Cl_2_), 7.36 (t, *J* = 8.1 Hz, 1H, *p*-H- C_6_H_3_Cl_2_), 6.18 (s, 2H, NH_2_), 4.60 (dd, *J* = 13.4, 9.1 Hz, 1H, C_6_-H), 2.86 (dd, *J* = 15.8, 9.1 Hz, 1H, C_5_-H), and 2.70 (dd, *J* = 15.9, 13.4 Hz, 1H, C_5_-H). ^13^C-NMR (100.5 MHz, DMSO-d*_6_*) δ: 169.6, 153.8, 135.6, 135.2, 134.8, 129.7, 129.6, 128.3, 84.9, 43.3, and 24.2. HRMS (70 eV, ESI) (m/z) calculated for C_19_H_11_D_5_Cl_2_N_5_O: 405.1046, [M + H]^+^; found: 405.1020, [M + H]^+^.

Synthesis of 4-amino-6-(2,6-dichlorophenyl)-2-(phenyl-*d_5_*)amino)-5,8-dihydropyrido[2,3-*d*]pyrimidin-7(*6H*)-one (**8**):

A quantity of 156.4 mg (0.4 mmol) of **7** was mixed with 5.6 mL of anhydrous MeOH in a 5 mL microwave vial and 20.8 mg (0.4 mmol) of NaOMe was added. The reaction mixture was heated in an oil bath at 140 °C for 40 min. Then, the solid was filtered, washed with water and diethyl ether, and dried overnight, to afford 121.3 mg (0.4 mmol, 78%) of **8** as a pale grey solid. mp: >300 °C. ^1^H-NMR (400 MHz, DMSO-d*_6_*) δ: 10.3 (s, 1H, N-H), 8.8 (s, 1H, NH-C_6_D_5_), 7.5 (dd, *J* = 13.5, 8.1 Hz, 2H, *m*-H- C_6_H_3_Cl_2_), 7.4 (t, *J* = 8.1 Hz, 1H, *p*-H- C_6_H_3_Cl_2_), 6.4 (s, 2H, NH_2_), 4.7 (dd, *J* = 13.1, 8.9 Hz, 1H, C_6_-H), 2.9 (dd, *J* = 15.8, 8.9 Hz, 1H, C_5_-H), and 2.8 (dd, *J* = 15.7, 13.2 Hz, 1H, C_5_-H). ^13^C-NMR (100.5 MHz, DMSO-d*_6_*) δ: 169.5, 161.5, 158.4, 155.8, 141.3, 135.7, 135.3–134.9, 129.9, 128.4, 84.4, 43.3, and 23.5. HRMS (70 eV, ESI) (m/z) calculated for C_19_H_11_D_5_Cl_2_N_5_O: 405.1046, [M + H]^+^; found: 405.1020, [M + H]^+^.

Synthesis of 4-amino-6-(2,6-dichlorophenyl)-2-((phenyl-*d_5_*)amino)pyrido[2,3-*d*]pyrimidin-7(*8H*)-one (**9**):

A quantity of 111.1 mg (0.3 mmol) of **8** was dissolved in 5 mL of anhydrous DMSO in a 10 mL round-bottom flask, and then, 30 mg (0.8 mmol) of NaH (60% in mineral oil) was added. An anhydrous CaCl_2_ tube was added on top of the round-bottomed flask to protect the content from moisture. The reaction mixture was stirred at room temperature for 10 min, then heated at 100 °C for 4 h. Then, the mixture was cooled down to room temperature and was diluted with 100 mL of water, forming a dark brown suspension. AcOH was added dropwise to the suspension until a neutral pH was reached. A heavy flocculation and a change of color from dark brown to pale brown were observed. The solid was filtered, washed with EtOH and EtOEt, and dried under vacuum overnight, affording 87.3 mg (0.2 mmol, 79%) of **9** as a pale brown solid. mp: >300 °C. ^1^H-NMR (400 MHz, DMSO-d*_6_*) δ: 11.8 (s, 1H, N-H), 9.3 (s, 1H, NH-C_6_D_5_), 8.1 (s, 1H, C_5_-H), 7.6–7.5 (dd, 2H, *m*-H- C_6_H_3_Cl_2_), 7.5–7.4 (t, 1H, *p*-H- C_6_H_3_Cl_2_), and 7.31 (s, 1H, NH_2_). ^13^C-NMR (100.5 MHz, DMSO-d*_6_*) δ: 161.7, 161.5, 159.9, 156.6, 140.9, 135.9, 135.8, 135.7, 130.7, 128.5, 122.0, and 91.7. HRMS (70 eV, ESI) (m/z) calculated for C_19_H_9_D_5_Cl_2_N_5_O: 403.0889, [M + H]^+^; found: 403.0876, [M + H]^+^.

Synthesis of 4-amino-6-(2,6-dichlorophenyl)-8-methyl-2-((phenyl-*d_5_*)amino)pyrido[2,3-*d*]pyrimidin-7(*8H*)-one (**IQS016-*d_5_***):

A quantity of 60.0 mg (0.15 mmol) of **9** was dissolved in 3.5 mL of anhydrous DMSO in a 10 mL round-bottom flask, and then, 5.97 mg (0.15 mmol) of NaH (60% in mineral oil) was added. An anhydrous CaCl_2_ tube was added on top of the round-bottom flask to protect the content from moisture. The reaction mixture was stirred at room temperature for 1 h before the addition of 12.5 µL (0.2 mmol) of MeI. The reaction mixture was stirred at room temperature and left overnight. After the reaction time, the mixture was diluted with 100 mL of water and a dark green suspension was formed. AcOH was added dropwise to the suspension until a neutral pH was reached. A heavy flocculation and a change of color were observed. The suspension was filtered, washed with EtOH and EtOEt, and dried under vacuum overnight, affording 42.5 mg (0.1 mmol, 68%) of **IQS016-*d_5_*** as a pale white solid. mp: >300 °C. ^1^H-NMR (400 MHz, DMSO-d*_6_*) δ: 9.4 (s, 1H, N_9_-H), 8.1 (s, 1H, C_5_-H), 7.6 (dd, 2H, C_15_-H), 7.5–7.3 (m, 3H, C_16_-H, N_12_-H_2_), and 3.61 (s, 3H, C_17_-H_3_). ^13^C-NMR (100.5 MHz, DMSO-d*_6_*) δ: 162.1, 160.9, 159.5, 156.5, 140.7, 135.8, 135.7, 134.4, 130.7, 128.5, 120.5, 92.1, and 28.8. HRMS (70 eV, ESI) (m/z) calculated for C_20_H_10_D_5_Cl_2_N_5_O: 417.1046, [M + H]^+^; found: 417.1032, [M + H]^+^.

### 3.2. Study of the Metabolism of **IQS016** and **IQS016-d_5_**

In vitro *Liver Microsomal Stability* [28,29]. A stock solution of the drug at 10 mM in DMSO (HPLC/MS grade, Sigma-Aldrich (Merck Life Sciences SLU, Madrid, Spain)) was prepared by weighting 1–2 mg of the compound under study in an Eppendorf tube using a high-precision analytical balance (M5000P, Sartorius). For each incubation trial, we placed 713 µL of deionized water, 200 µL of phosphate buffer saline (PBS, pH = 7.4), 50 µL of NADPH solution A (Corning^®^ Gentest™ NADPH Regenerating System Solution A, ref. 451220), 10 µL of NADPH of solution B (Corning^®^ Gentest™ NADPH Regenerating System Solution B, ref. 451200), and 2 µL of drug stock solution into a 1.5 mL Eppendorf (each compound was prepared in duplicate). We incubated each sample at 37 °C for 5 min in an Eppendorf ThermoMixer. The reaction was initiated by the addition of 25 µL of pooled male rat-liver microsomes (Corning^®^ Gentest™ Rat Liver Microsomes Pooled Male (Sprague–Dawley), 20 mg/mL, 452501). At the desired time points (0, 5, 15, 30, and 60 min), we transferred 100 µL of the sample into a vial containing 100 µL of acetonitrile (HPLC/MS-grade, VWR) in ice. We centrifuged each quenched sample at 3000 rpm for 5 min or until there was a contained pellet at the bottom of each tube. We transferred the supernatant to an HPLC vial. We analyzed the metabolic profile using HPLC-MS/MS equipment. We monitored the parent compound at the different time points: 0, 5, 15, 30, and 60 min. We converted data to % of remaining compound by dividing by the time-zero concentration value. We established the masses of the potential metabolites for identification.

*Chromatographic Method*—Equipment: Exion 1.0 AD coupled to an X500B QTOF System, SCIEX. Column: Phenomenex Luna Omega 1.6 mm Polar C18 (100 × 2.1 mm). Injection volume: 3 µL. Flow rate: 0.4 mL/min. T_column_: 40 °C. T_sampler_: 15 °C. Gradient: see Table 2.

Detection—Mode: SWATH scan with a combination of a full-scan TOF spectrum (100–1000 Da). Ion source: Turbo Electro Spray (+). Curtain gas: 35 psi. CAD gas: 9. Ion source gas 1: 60 psi. Ion source gas 2: 60 psi. Temperature: 500 °C. Spray voltage: 5500 V. Declustering potential: 80 V. Collision energy: 10 V. CID parameters: Collision energy: 45 V. Collision energy spread: 25 V.

### 3.3. Kinase Inhibition Profile of **IQS016** and **IQS016-d_5_**

The kinase inhibition profiles of compounds were evaluated at Reaction Biology (https://www.reactionbiology.com/, accessed on 19 December 2024) by measuring residual activity values at a concentration of 10 µM of the test compound in singlicate in front of the following kinases: Btk, Lyn, and Syk aa1-635. We used the following protocol: the compounds were dissolved to 1 × 10^−3^ M stock solutions in 100% DMSO. Subsequently, 100 μL of each stock solution was transferred into wells A3-F12 of a microtiter plate (“master plate”). Wells A1-F2 were filled with 100 μL 100% DMSO as controls. A total of 5 × 10 μL of the master plates were aliquoted into 5 copy plates, which were stored at -20 °C until use. For the testing of each group of up to 8 kinases, one copy plate was used. In the process, 90 μL H_2_O was added to each well of a copy plate. To minimize precipitation, the H_2_O was added to each well only a few minutes before the transfer of the compound solutions into the assay plates. Each plate was shaken thoroughly, resulting in a “compound dilution plate” with a compound concentration of 1 × 10^−4^ M/10% DMSO. This plate was used for the transfer of 5 μL compound solution into the assay plates. The final volume of the assay was 50 μL. All compounds were tested at 1 × 10^−5^ M in singlicate. The final DMSO concentration in the reaction cocktails was 1% in all cases. The compound dilution plates were disposed of at the end of each working day.

A radiometric protein kinase assay (^33^PanQinase^®^ Activity Assay) was used for measuring the kinase activity of the corresponding protein kinases. All kinase assays were performed in 96-well FlashPlatesTM from Perkin Elmer (Boston, MA, USA) in a 50 μL reaction volume. The reaction cocktail was pipetted in 4 steps in the following order: 10 μL of non-radioactive ATP solution (in H_2_O); 25 μL of assay buffer/[γ-33P]-ATP mixture; 5 μL of test sample in 10% DMSO; 10 μL of enzyme/substrate mixture. The assay for all protein kinases contained 70 mM HEPES-NaOH of pH 7.5, 3 mM MgCl_2_, 3 mM MnCl_2_, 3 μM Na-orthovanadate, 1.2 mM DTT, ATP (variable amounts, corresponding to the apparent ATP-Km of the respective kinase), [γ-^33^P]-ATP (approx. 8 × 10^5^ cpm per well), protein kinase (variable amounts), and substrate (variable amounts). The protein kinase reaction cocktails were incubated at 30 °C for 60 min. The reaction was stopped with 50 μL of 2% (*v*/*v*) H_3_PO_4_; plates were aspirated and washed two times with 200 μL0.9 % (*w*/*v*) NaCl. All assays were performed with a BeckmanCoulter Biomek 2000/SL robotic system. The incorporation of ^33^Pi (counting of “cpm”) was determined with a microplate scintillation counter (Microbeta, Wallac). All protein kinase assays were performed with a BeckmanCoulter Core robotic system.

For each kinase, the median value of the cpm of six wells of column 1 of each assay plate was defined as “low control” (n = 6). This value reflected the unspecific binding of radioactivity to the plate in the absence of a protein kinase but in the presence of the substrate. Additionally, for each kinase, the median value of the cpm of six wells of column 2 of each assay plate was taken as the “high control”, i.e., full activity in the absence of any inhibitor (n = 6). The difference between high and low controls of each enzyme was taken as 100% activity. As part of the data evaluation, the low control of each kinase was subtracted from the high control value, as well as from their corresponding “compound values”. The residual activity (in %) for each compound well was calculated by using Equation (1):

Res. Activity (%) = 100 × [(signal of the compound − low control)/(high control − low control)](1)

As a parameter for assay quality, the Z′-factor [34] for the low and high controls of each assay plate (n = 8) was used. Reaction Biology’s criterion for repetition of an assay plate is a Z′-factor below 0.4 [35]. Z′-factors did not drop below 0.51, indicating an excellent assay quality.

### 3.4. D In Vitro Biological Testing of **IQS016** and **IQS016**-d_5_

A stock solution of the compound in dimethyl sulfoxide (DMSO, Sigma-Aldrich) was prepared at a sufficiently high concentration to minimize the presence of DMSO in the cell culture. In this work, the maximum concentration of DMSO used was 0.02%. Cells were cultured on 25 cm^3^ plastic flasks. PANC-1 (human pancreatic adenocarcinoma, ATCC number CRL-1469) and hNDF (primary human normal dermal fibroblast, ATCC number OCS-201-012) cells were cultivated with DMEM (Dulbecco’s Modified Eagle’s medium low glucose, Gibco) while BxPC-3 (human pancreatic adenocarcinoma, ATCC number CRL-1687) cells were incubated with RPMI 1640 (Roswell Park Memorial Institute’s medium containing 11.2 mmol/L glucose, Thermo Fisher). Both culture media were supplemented with 10% (*v*/*v*) FBS (fetal bovine serum, Thermo Fisher), 1% (*v*/*v*) L-glutamine (Thermo Fisher), and 1% (*v*/*v*) penicillin–streptomycin (Thermo Fisher). All experiments were performed with passages between 5 and 18. Cells were maintained at 37 °C in a humidified incubator with 5% CO_2_. When 40–50% of confluence was reached, the cells were seeded on 48-well plates: 10,000 cells/well. Seed n = 3 wells/condition (cell line and drug concentration). After 24 h, the culture medium was removed and fresh medium supplemented with the drug at the desired concentration (0.01, 0.1, 1, and 10 mM) was added. In parallel, a control (medium) and placebo solution (maximum DMSO concentration) were tested to demonstrate that the presence of DMSO did not induce significant cell death. The cells were incubated with the drug for 72 h at 37 °C in a humidified incubator with 5% CO_2_. An MTT (3-[4,5-dimethylthiazol-2-yl]-2,5-dipheniltetrazolium bromide, Sigma-Aldrich) stock solution at 10 mg/mL in PBS 1X (Phosphate buffer saline, Gibco (Thermo Fisher Scientific SLU, Alcobendas, Spain)) was prepared and filtered through 0.22 mm filter. The resulting solution was stored between 2 and 8 °C until the day of the experiment. On the day of the experiment (after 72 h of drug incubation), the MTT stock solution was diluted with culture medium at a final concentration of 0.5 mg/mL. The culture medium supplemented with the drug was removed and the MTT 0.5 mg/mL solution was added. The samples were incubated for 3 h at 37 °C and protected from light. At the end of the incubation period, the MTT solution was removed and the formazan crystals were solubilized with 200 mL of DMSO. We pipetted them up and down to lyse cells and helped solubilize the compound. We read the absorbance using 570 nm Infinite^®^ 200 PRO instrument (Tecan Life Science (Tecan Ibérica Instrumentación SL, Barcelona, Spain)).

Data analysis: The raw data were imported to an Excel sheet and the absorbance values of the samples were corrected by extracting the solvent absorbance (DMSO) at 570 nm. The data were converted to % of cell viability by normalizing them with the absorbance of the control sample at 570 nm. The % of cell viability was plotted against concentration to derive the IC_50_ of the drug.

## 4. Conclusions

**IQS016-*d_5_*** was synthesized from aniline-2,3,4,5,6-*d_5_* to improve the metabolic stability of **IQS016** in front of rat-liver microsomes, showing that the deuterated compound was by far more stable than the 2-phenylamino derivative, thus proving the benefits of deuteration to increase metabolic stability.

Subsequently, we compared the inhibitory activity (evaluated against a group of selected isolated TK receptors) and the in vitro biological activity (in 2D cultures of pancreatic cancer cell lines PANC-1 and BxPC-3 and normal dermal fibroblasts, hNDF) of **IQS016** and **IQS016-*d_5_***. Although a clear improvement in the inhibitory activity of **IQS016-*d_5_*** was observed, contrary to our expectations, the % of cell viability was not reduced accordingly but, on the contrary, **IQS016** showed a better efficacy against the pancreatic cancer cell lines. This result indicates that the substitution of hydrogen atoms by deuterium atoms in a molecule may be beneficial from the point of view of metabolic stability but not necessarily from the biological point of view.

## Figures and Tables

**Table 1:** Results of **IQS016** and **IQS016-*d_5_*** evaluated against a group of selected TK receptors obtained at Reaction Biology (low residual activities indicate better inhibitory activity) ^1^.

Comp.	BTK	EGFR	FGFR1	FGFR2	IGF1R	JAK3	PDGFR	VEGFR2
**IQS016-*d_5_***	7	5	12	7	28	61	44	29
**IQS016**	21	11	21	18	42	70	61	53

^1^ Residual activity on isolated proteins: less than 20% is shown in green, between 20–60% in yellow, and greater than 60% in red.

**Table 2:** Gradient.

Time (min)	%A (ACN[H^+^])	B (H_2_O[H^+^])
0.00	90.0	10.0
1.00	90.0	10.0
9.00	1.0	99.0
11.00	1.0	99.0
11.10	90.0	10.0
15.00	90.0	10.0

## Data Availability

The original contributions presented in the study are included in the article/Supplementary Materials; further inquiries can be directed to the corresponding author/s.

## Supplementary Materials

The following supporting information can be downloaded at: https://www.mdpi.com/article/10.3390/molecules29246042/s1, NMR spectra of the compounds synthesized; Table S1. Metabolic stability of IQS016 and d5-IQS016; Table S2. Cell viability of PANC-1, BxPC-3, and hNDF with IQS016 and d5-IQS016.
