# Supplementary material for: The Use of a Penta-Deuterophenyl Substituent to Improve the Metabolic Stability of a Tyrosine Kinase Inhibitor
Source: Molecules. 2024 Dec 22;29(24):6042. doi: 10.3390/molecules29246042 (PMC11679266; doi:10.3390/molecules29246042)
Supplement: Supplementary file 1 [file molecules-29-06042-s001.zip › molecules-3325944-supplementary.pdf]

SUPPLEMENTARY INFORMATION

Use of a penta-Deuterophenyl Substituent to Improve the Metabolic Stability of a Tyrosine Kinase Inhibitor

Júlia Dulsat, Raimon Puig de la Bellacasa and José I. Borrell *

Grup de Química Farmacèutica, IQS School of Engineering, Universitat Ramon Llull, Via Augusta, 390, E-08017 Barcelona, Spain. E-mail: jose.borrell@iqs.url.edu

Table of Contents

	Page
NMR Spectra	2
Table S1. Metabolic stability of **IQS016** and ***d*$_5$-IQS016**	7
Table S2. Cell viability of PANC-1, BxPC-3, and hNDF with **IQS016** and ***d*$_5$-IQS016**	7

^{1}H-NMR spectrum (DMSO-d_6) of aniline-2,3,4,5,6-d_5 (**2**)

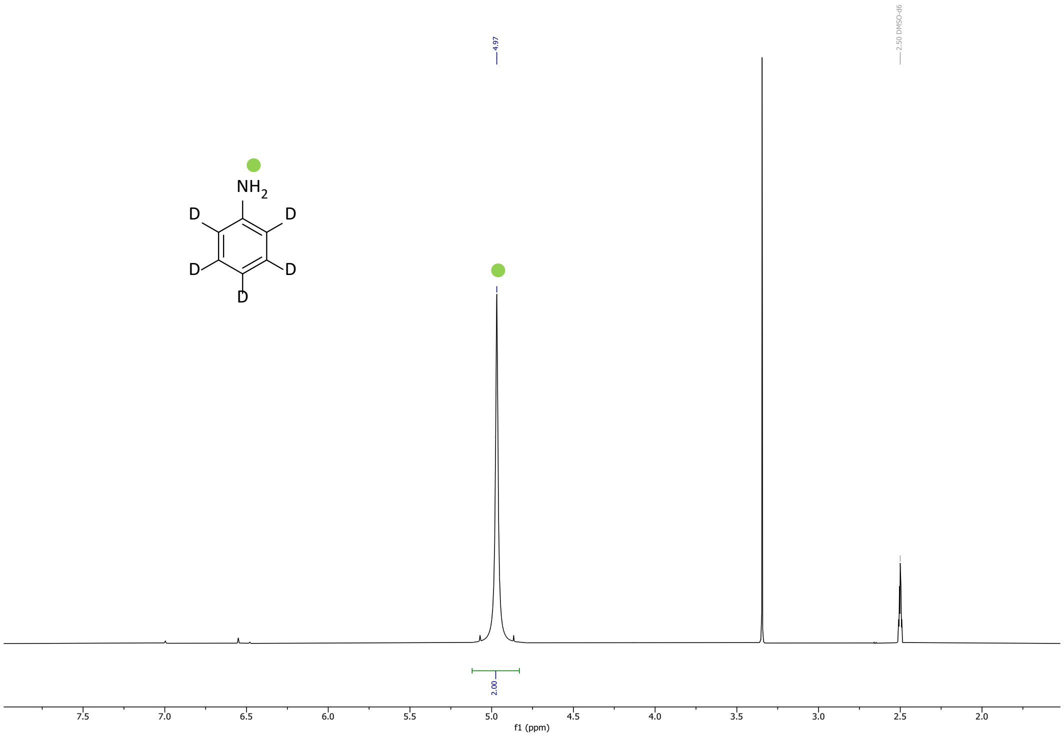

^{13}C-NMR spectra (D_2O) of aniline-2,3,4,5,6-d_5 (**2**) and 1-(phenyl-d_5)guanidine hydrogen sulfite (**3**)

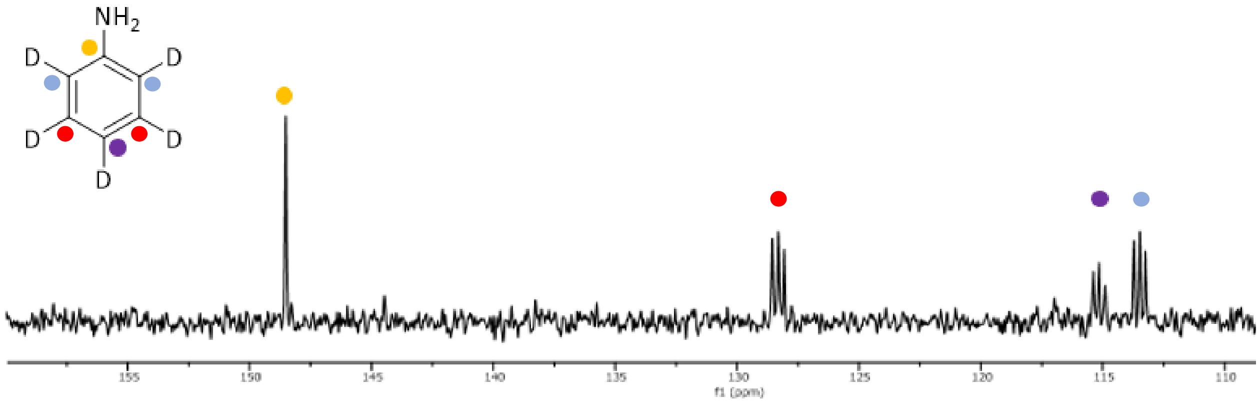

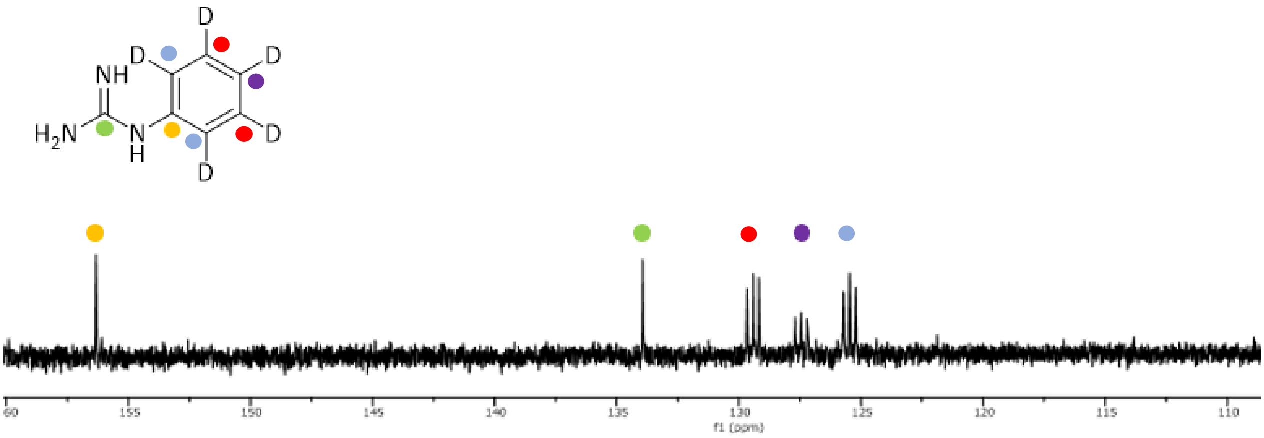

^{1}H-NMR spectrum (DMSO-*d*$_6$) of 2-amino-6-(2,6-dichlorophenyl)-4-imino-3-(phenyl-*d*$_5$)-4,5,6,8-tetrahydropyrido[2,3-*d*]pyrimidin-7(*3H*)-one (**7**)

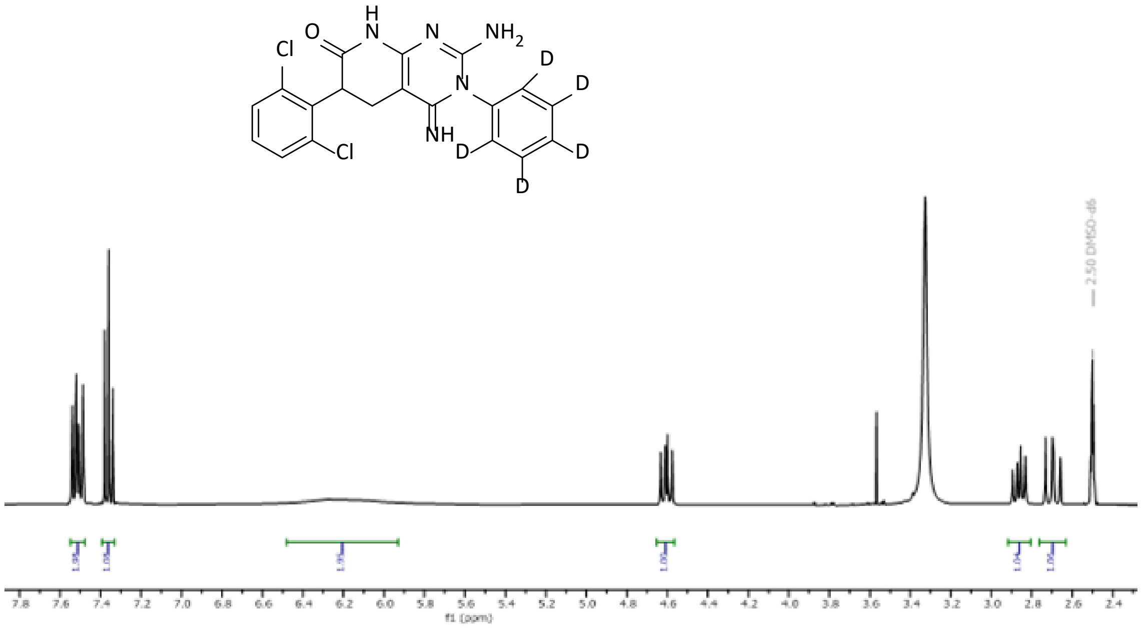

^{13}C-NMR spectrum (DMSO-*d*$_6$) of 2-amino-6-(2,6-dichlorophenyl)-4-imino-3-(phenyl-*d*$_5$)-4,5,6,8-tetrahydropyrido[2,3-*d*]pyrimidin-7(*3H*)-one (**7**)

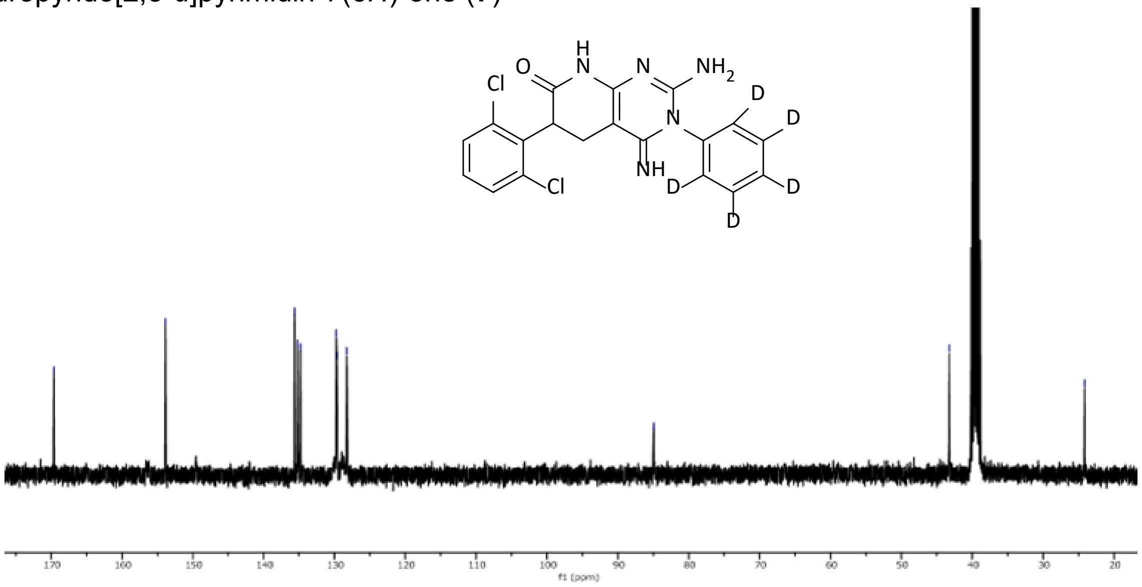

^{1}H-NMR spectrum (DMSO-d_6) of 4-amino-6-(2,6-dichlorophenyl)-2-(phenyl-d_5)amino)-5,8-dihydropyrido[2,3-*d*]pyrimidin-7(*6H*)-one (**8**)

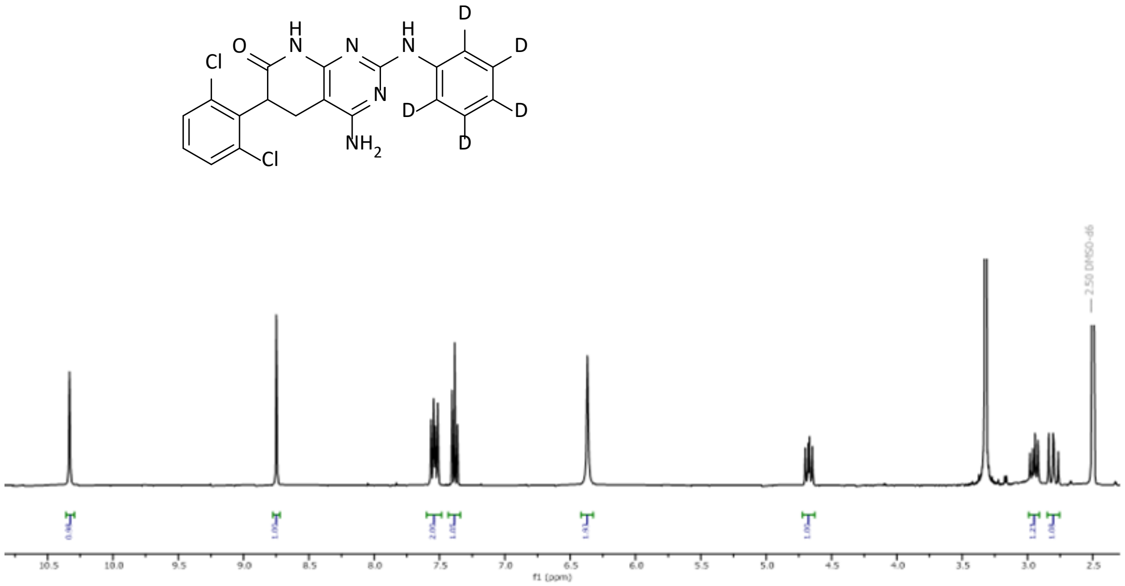

^{13}C-NMR spectrum (DMSO-d_6) of 4-amino-6-(2,6-dichlorophenyl)-2-(phenyl-d_5)amino)-5,8-dihydropyrido[2,3-*d*]pyrimidin-7(*6H*)-one (**8**)

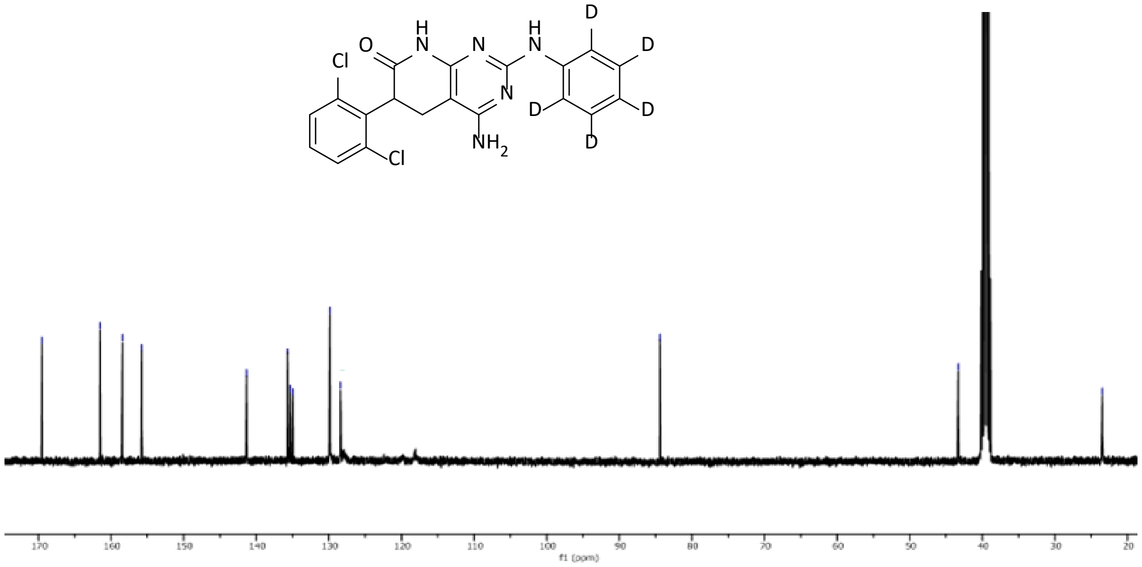

^{1}H-NMR spectrum (DMSO-d_6) of 4-amino-6-(2,6-dichlorophenyl)-2-((phenyl-d_5)amino)pyrido[2,3-*d*]pyrimidin-7(*8H*)-one (**9**)

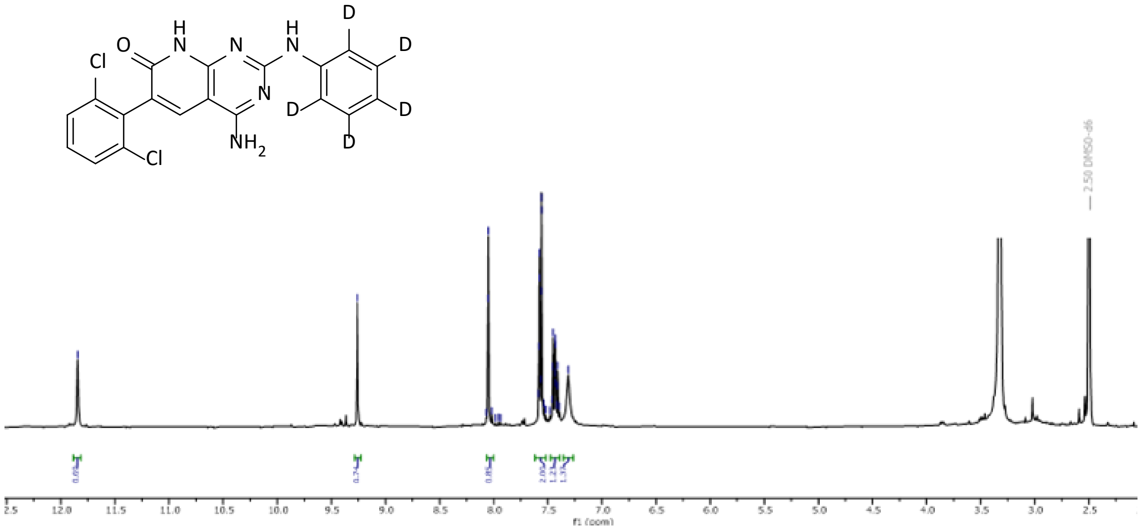

^{13}C-NMR spectrum (DMSO-d_6) of 4-amino-6-(2,6-dichlorophenyl)-2-((phenyl-d_5)amino)pyrido[2,3-*d*]pyrimidin-7(*8H*)-one (**9**)

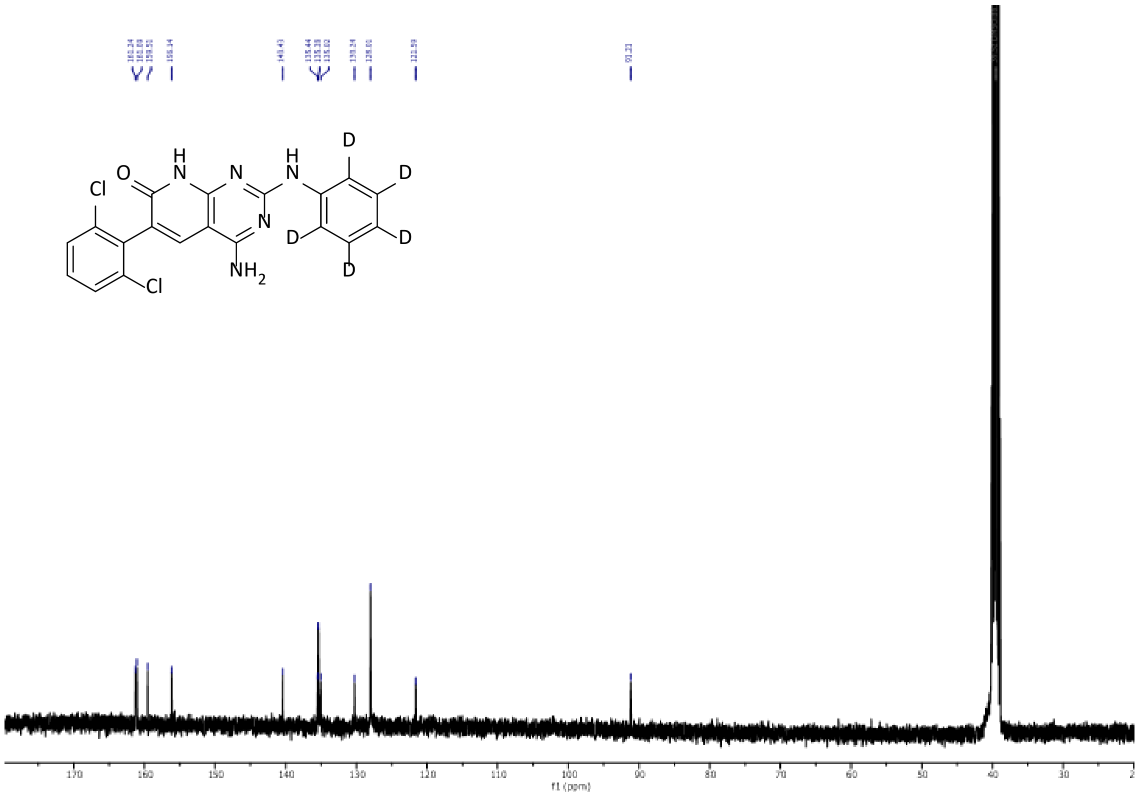

^{1}H-NMR spectrum (DMSO-d_6) of 4-amino-6-(2,6-dichlorophenyl)-8-methyl-2-((phenyl-d_5)amino)pyrido[2,3-*d*]pyrimidin-7(*8H*)-one (**IQS016-*d*$_5$**)

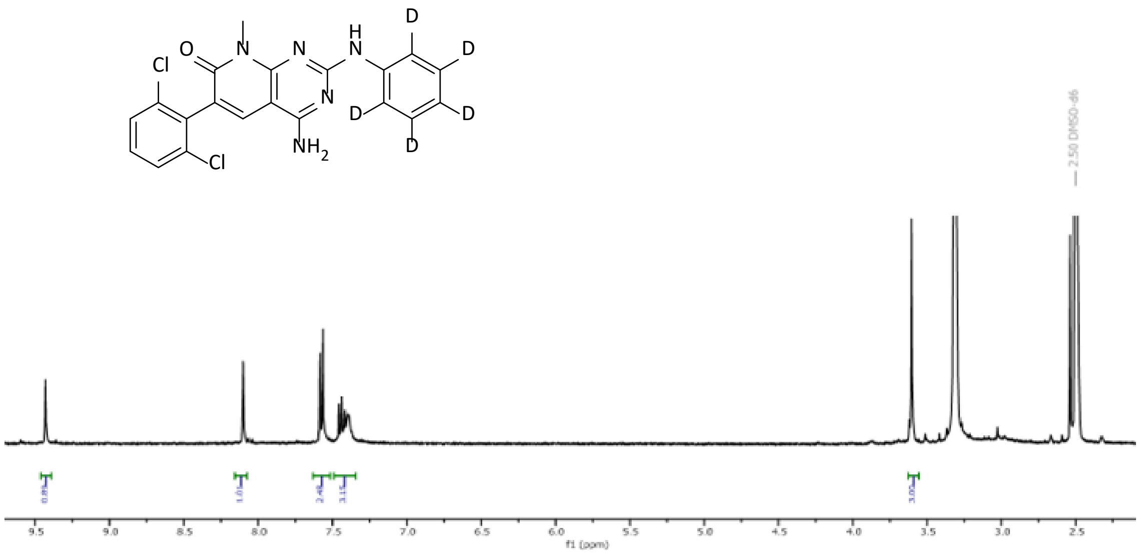

^{13}C-NMR spectrum (DMSO-d_6) of 4-amino-6-(2,6-dichlorophenyl)-8-methyl-2-((phenyl-d_5)amino)pyrido[2,3-*d*]pyrimidin-7(*8H*)-one (**IQS016-*d*$_5$**)

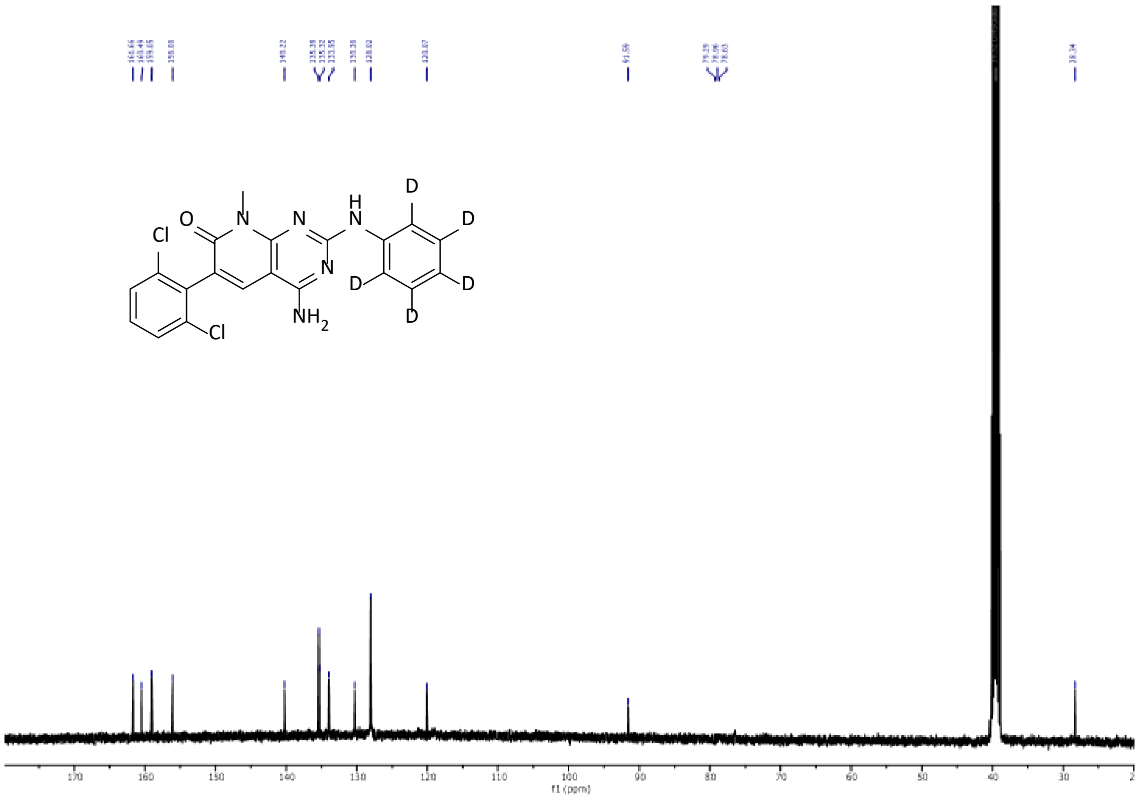

Table S1. Metabolic stability of IQS016 (n=2) and IQS016-d_5 (n=2) using the rat liver microsomes incubation protocol.

Time (min)	IQS016		IQS016-d_5	
0	100	100	100	100
5	66	80	97	92
15	48	68	88	83
30	39	52	79	64
60	34	49	85	77

Table S2. Cell viability (%) in a 2D culture of PANC-1, BxPC-3, and hNDF (purple) after 72 h of incubation with IQS016 and IQS016-d_5 (n=3).

IQS016

Concentration (μM)	PANC-1			BxPC-3			hNDF		
0.01	97	63	82	53	78	65	58	65	60
0.1	88	66	88	41	64	50	72	65	69
1	106	72	91	57	41	43	71	70	75
10	66	76	63	57	44	44	73	71	68

IQS016-d_5

Concentration (μM)	PANC-1			BxPC-3			hNDF		
0.01	85	85	80	37	22	58	68	68	50
0.1	95	106	90	42	40	57	54	65	55
1	77	86	80	28	22	52	63	74	50
10	35	36	37	10	8	8	32	38	35
